# Oxytocin and arginine vasopressin receptor evolution: implications for adaptive novelties in placental mammals

**DOI:** 10.1590/1678-4685-GMB-2015-0323

**Published:** 2016-08-08

**Authors:** Pamela Paré, Vanessa R. Paixão-Côrtes, Luciana Tovo-Rodrigues, Pedro Vargas-Pinilla, Lucas Henriques Viscardi, Francisco Mauro Salzano, Luiz E. Henkes, Maria Catira Bortolini

**Affiliations:** 1 Programa de Pós-Graduação em Genética e Biologia Molecular, Departamento de Genética, Universidade Federal do Rio Grande do Sul (UFRGS), Porto Alegre, RS, Brazil; 2 Programa de Pós-Graduação em Genética e Biodiversidade, Instituto de Biologia, Universidade Federal da Bahia (UFBA), Salvador, BA, Brazil.; 3 Laboratório de Fisiologia da Reprodução Animal, Universidade Federal de Santa Catarina (UFSC), Curitibanos, SC, Brazil.; 4 Programa de Pós-Graduação em Epidemiologia, Universidade Federal de Pelotas (UFPEL), Pelotas, RS, Brazil.

**Keywords:** Oxytocin receptor, Arginine vasopressin receptors, molecular evolution, protein disorder, interaction motifs

## Abstract

Oxytocin receptor (*OXTR*) and arginine vasopressin receptors (*AVPR1a, AVPR1b*, and *AVPR2*) are paralogous genes that emerged through duplication events; along the evolutionary timeline, owing to speciation, numerous orthologues emerged as well. In order to elucidate the evolutionary forces that shaped these four genes in placental mammals and to reveal specific aspects of their protein structures, 35 species were selected. Specifically, we investigated their molecular evolutionary history and intrinsic protein disorder content, and identified the presence of short linear interaction motifs. *OXTR* seems to be under evolutionary constraint in placental mammals, whereas *AVPR1a, AVPR1b*, and *AVPR2* exhibit higher evolutionary rates, suggesting that they have been under relaxed or experienced positive selection. In addition, we describe here, for the first time, that the OXTR, AVPR1a, AVPR1b, and AVPR2 mammalian orthologues preserve their disorder content, while this condition varies among the paralogues. Finally, our results reveal the presence of short linear interaction motifs, indicating possible functional adaptations related to physiological and/or behavioral taxa-specific traits.

## Introduction

Genome and tandem duplication events have an important role in biological evolution (Van de Peer *et al.*, 2009). These processes give rise to paralogous genes, which can evolve by speciation along the evolutionary timeline, thus giving rise to orthologous genes (Fitch, 1970; Gabaldón and Koonin, 2013). As result of these processes, so-called "gene families" emerge, whose members may retain similar or identical functions, but might also diverge extensively, resulting in adaptive novelties (Neduva and Russell, 2005; Huang and Sarai, 2012). The emergence and differentiation of the paralogous neuroendocrine nonapeptides oxytocin (OXT) and vasopressin (AVP; Gwee *et al.*, 2009), as well of their paralogous receptors (OXTR and AVPR1a, AVPR1b, AVPR2, respectively) illustrate this phenomenon (Yamaguchi *et al.*, 2012; Lagman *et al.*, 2013).

The OXT peptide is comprised of a nine amino acid sequence (Lee *et al.*, 2009), differing in only two amino acids from its paralogue, AVP. These nonapeptides, produced in their highest quantities in the brain, mediate both similar and distinct functions through their interactions with their native receptors (OXTR; AVPR1a, AVPR1b, and AVPR2), which are produced in various organs and tissues (Barberis *et al.*, 1998). Some level of cross-reaction among OXT and AVP with their non-native receptors occurs as well, but with distinct affinities (Zingg and Laporte, 2003; Slusarz *et al.*, 2013). For instance, the synthesis of OXTR in the uterus and mammary glands guarantees uterine contraction and milk ejection in placental mammals (Kimura *et al.*, 1998; Gimpl and Fahrenholz, 2001), whereas AVPR1a mediates vasoconstriction, AVPR1b promotes the release of adrenocorticotropic hormone, and AVPR2 mediates water homeostasis (Koshimizu *et al.*, 2012). In the brain, these four receptors promote the functions of OXT and AVP associated with complex behaviors (Koshimizu *et al.*, 2012; Koehbach and Gruber, 2013). The presence of this interconnected system throughout the animal kingdom indicates that the typical roles of these receptors in placental mammals are likely exaptations of ancient functions, such as regulation of fluid balance and egg-laying (Oumi *et al.*, 1996; Fujino *et al.*, 1999).

Orthologues of *OXTR, AVPR1a*, *AVPR1b*, and *AVPR2* have been described in all vertebrates investigated to date (Gwee *et al.*, 2009; Lagman *et al.*, 2013). It has been proposed that *AVPR1a, AVPR1b*, and *OXTR* originate from a common ancestral gene, whereas *AVPR2* originates from another ancestral gene (Lagman *et al.*, 2013). Functionally, the AVPR2 present in placental mammals differs from AVPR1a, AVPR1b, and OXTR, since it activates adenylatecyclases instead of phospholipases to interact with G-proteins (Liu and Wess 1996; Ocampo *et al.*, 2012). This receptor gene family emerged in the two rounds of whole vertebrate genome duplication that occurred immediately prior to or during the Cambrian era, similar to innumerous other gene families found in the vertebrate genomes (Yamaguchi *et al.*, 2012; Lagman *et al.*, 2013). However, in fishes and amphibians, additional *AVPR2* subtypes can be found as well (Yamaguchi *et al.*, 2012; Lagman *et al.*, 2013). OXTR, AVPR1a, AVPR1b, and AVPR2, along with other similar receptors, belong to class 1 G protein-coupled receptors (GPCRs), being composed of four extracellular regions (N-terminal; ECL1–3), seven transmembrane regions (TM1–7), and four intracellular regions (ICL1–3; C-terminal).

Despite some remarkable and taxa-specific variation in OXT and AVP observed in placental mammals (Lee *et al.*, 2011; Stoop, 2012; Koehbach and Gruber, 2013; Ren *et al.*, 2015; Vargas-Pinilla *et al.*, 2015), the ability of the OXT/AVP system to evolve (evolvability; Pigliucci 2008; Wagner 2008) is known to be mediated primarily by changes in their respective receptors. Previously, we described several inter and intraspecific putative functional variants in the regulatory and coding regions of these receptors (Vargas-Pinilla *et al.*, 2015). We also demonstrated that some OXTR variants are clearly co-evolving with the OXT forms found in New World monkey (NWm) species (Vargas-Pinilla *et al.*, 2015).

Changes in amino acid sequence might have several implications for protein structure. For instance, it is known that GPCRs have long intrinsically disordered regions (IDRs; Jaakola *et al.*, 2005; Tovo-Rodrigues *et al.*, 2014). IDRs have a central role in the regulation of signaling pathways and in crucial cellular processes, including the regulation of transcription and translation, and acting also as hubs (highly connected proteins; Wright and Dyson, 2014; and references therein). The primary feature of IDRs is the ability to assume different conformations that allow interaction with multiple partners; *i.e*., IDRs lack a stable three-dimensional structure (Uversky, 2015). Previous studies showed that GPCRs have greater intrinsic disorder content in N-terminal, ICL3, and C-terminal regions, which are important for interactions with other molecules (Jaakola *et al.*, 2005; Tovo-Rodrigues *et al.*, 2014). Short linear motifs (SLiMs) are common elements in IDRs and consist of approximately 3–11 contiguous amino acids, of which usually two or three are functionally important (Neduva and Russell, 2005; Dinkel *et al.*, 2014). As a consequence, just a few amino acid changes can result in an alteration from an inert stretch into a functional interactive sequence, or vice-versa, thereby providing extraordinary evolutionary plasticity (Neduva and Russell, 2005). Thus, SLiMs probably play a significant role in the functioning of OXTR, AVPR1a, AVPR1b, and AVPR2.

Our goal in this study was to elucidate the evolutionary forces that shaped the four paralogous genes *OXTR, AVPR1a*, *AVPR1b*, and *AVPR2* and to reveal specific aspects of the receptor structures using a set of 35 placental mammals. Since OXTR, AVPR1a, AVPR1b, and AVPR2 are GPCRs, we also predicted their intrinsic disorder levels, as well as the presence of putative SLiMs in sites located at IDR, which have a high probability of being under positive selection or relaxed functional constraint for the regions containing high disorder levels. Finally, we aimed to evaluate whether the identified structural changes might have functional implications, so that they could be associated with the adaptive novelties of placental mammals.

## Materials and Methods

### Data mining

A total of 35 species of placental mammals were analyzed considering the data available for the four genes *OXTR, AVPR1a*, *AVPR1b*, and *AVPR2* in the same organisms (Table S1). We opted to analyze orthologues within this group of organisms because better information is available for them than in other groups. Only species with available coding sequence of all paralogues were considered. Species with incomplete sequences or those missing any paralogue were excluded from the analysis. The full coding sequences considered as human orthologues were downloaded from ENSEMBL. Additionally, the known human *OXTR, AVPR1a*, *AVPR1b*, and *AVPR2* sequences were used as queries in Genomic BLAST, the UCSC Genome Browser database, and UniProt. Sequences were selected in each database according to best coverage, considering annotation exemplifying whether the sequence represented the canonic form or the major transcript (Table S1). The sequence alignments were performed using the MUSCLE algorithm (Edgar, 2004) included in the Mega 6.0 software package (Tamura *et al.*, 2013) and were manually reviewed. Additionally, all the alignments were submitted to the GUIDANCE web server for application of the MAFFT algorithm and were further checked by hand (OXTR, Supplementary Material 1, AVPR1a, Supplementary Material 2, AVPR1b, Supplementary Material 3, and AVPR2, Supplementary Material 4 (Penn *et al.*, 2010).

### Data analysis

Phylogenetic analysis was performed using the Maximum Likelihood method (Mega 6.0 version; Tamura *et al.*, 2013). The best-fit model (Jones-Taylor-Thornton matrix-based model +G +F) for protein evolution was selected using the Akaike Information Criterion (AIC) and the Bayesian Information Criterion (BIC) available in Mega (Jones *et al.*, 1992). A bootstrap support of 1000 replicates was used (Tamura *et al.*, 2013).

To detect positive selection, we carried out a molecular evolutionary analysis based on an inter-specific phylogenetic comparison of protein-coding genes. The distinct models and parameters used to test adaptive evolution at codon sites (NsSites test) were provided through the Phylogenetic Analysis by Maximum Likelihood package (PAML 4.7). This approach allows ω, the non-synonymous/synonymous rate ratio (dN/dS), to vary among sites in several different codon substitution models, where ω < 1 indicates negative selection, ω ≅ 1 indicates neutral or relaxed selection, and ω > 1 indicates positive selection. The species phylogenetic tree submitted to PAML was provided by Ensembl, and edited with PhyloWidget (Jordan and Piel, 2008), providing the unrooted tree. The tree topology formulation was carried out in accordance with the phylogenetic articles of primates (Perelman *et al.*, 2011) and mammals (Meredith *et al.*, 2011; Song *et al.*, 2012; Figure S1).

The neutral model (null) does not allow positive selection and was compared to the model that admits positive selection (alternative, ω > 1). This statistical comparison was performed using a likelihood ratio test (LRT) to infer the goodness-of-fit between the two models, wherein a relatively simpler model is compared to a more complex to verify if it fits a particular dataset significantly better. Because M1a (nearly neutral), M2a (Positive selection), M7 (beta), and M8 (beta& ω) are considered as the useful models, two LRTs were performed: M1a *vs* M2a and M7 *vs* M8 (Yang, 2007). In the first test, M1a, a neutral model that allows two categories of ω classes (ω0 < 1, and ω1 = 1), was compared to the M2a selection model that admits three ω classes, one of which might be a value > 1. In the second test, M7, a neutral model estimating a beta distribution with ten ω classes, was compared to a similar model, M8, which indicates positive selection with eleven ω classes, assuming a beta distribution and one class with ω > 1. A Bayesian approach is included within PAML to calculate the posterior probabilities of site classes of ω values. These probabilities were thus used to verify that sites had ω > 1. Bayes Empirical Bayes (BEB) was available for the models that admit positive selection (M2a and M8). It is worth noting that the alignment included sequences selected in each database according to the best coverage, and the gaps were removed using the option "cleandata = 1", which removes all sites with ambiguous characters. To check whether the gaps in the alignment would indicate an actual evolutionary change, we also performed an analysis considering these positions (cleandata option = 0). The sites found with a higher posterior probability to be under positive selection in BEB were further explored and all amino acid changes were categorized into classes of chemical similarity using the Grantham score (GS). The changes were classified as conservative (GS 0–50), moderately conservative (51–100), moderately radical (101–150), and radical (> 151; Grantham, 1974; Li *et al.*, 1984).

The protein intrinsic disorder contents of OXTR, AVPR1a, AVPR1b, and AVPR2 were estimated using the PONDR-FIT predictor (Xue *et al.*, 2013), a consensus artificial neural network (ANN) prediction method developed by combining the outputs of several individual disorder predictors. As output, this meta-predictor generates a single score per amino acid residue indicating the likelihood of its being structured or disordered. The threshold of 0.5 is used to classify the residue as ordered (below the threshold) or disordered (above the threshold; Xue *et al.*, 2013). The proportion of residues predicted as disordered in each protein domain was utilized to compare paralogue and orthologue receptor structure and flexibility. Thereafter, we predicted the secondary protein structure for each species' sequence using Psipred (Buchan *et al.*, 2013). The disorder proportion estimate for each domain was used to compare species for each paralogue as well as among paralogues considering the entire set of retrieved sequences, using Kruskal-Wallis and Mann-Whitney tests (Kruskal and Wallis, 1952). In addition, a Spearman test was used to test whether a correlation existed between the disorder values and the values of ω.

SLiMs located within the disordered regions of receptors were predicted using the Eukaryotic Linear Motif (ELM) web server (Dinkel *et al.*, 2014). Since these predictor analyses can introduce false positive results (Teyra *et al.*, 2012), we considered just SLiMs with experimental evidence provide by ELM.

## Results

### Phylogenetic analysis

**Figure 1:**
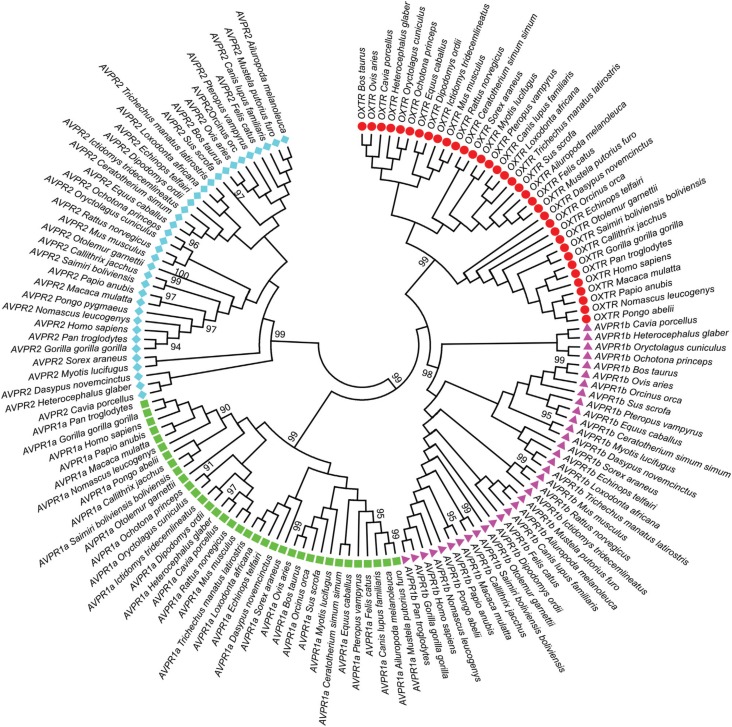
Molecular Phylogenetic analysis of OXTR, AVPR1a, AVPR1b, and AVPR2 by the maximum likelihood method (as described in Materials and Methods). The analysis involved 140 amino acid sequences. All positions with less than 95% site coverage were eliminated. There were a total of 317 positions in the final dataset.

Initially we performed a phylogenetic analysis of all four paralogue receptors through 35 placental mammalian species (Table S1). The maximum likelihood tree (Figure 1) presents well-defined clusters, separating the four genes with a good statistical support. The topology of the tree indicates that AVPR1a, AVPR1b, and OXTR form related clusters. AVPR2, on the other hand, seems to be more phylogenetically distant from the other three genes. These findings are in agreement with the hypotheses suggested by Lagman *et al.* (2013).

Notably, all the postulated orthologues clustered in their specific clades, whereas the phylogenetic relationships among them, in some cases, did not reproduce the expected phylogenetic relationships among species (Figure 1 and S1; see for example the *Myotis lucifugus*/microbat AVPR1b sequence, which is clustered with the *Dasypus novemcinctus*/armadillo AVPR1b sequence). These results indicated that the considered genes are really orthologues, in other words, the same genes in each different species. On the other hand, when inconsistencies between species and gene trees are detected, a simple neutral model of mutation and drift is insufficient to explain the observed pattern.

### Molecular evolutionary patterns

**Figure 2:**
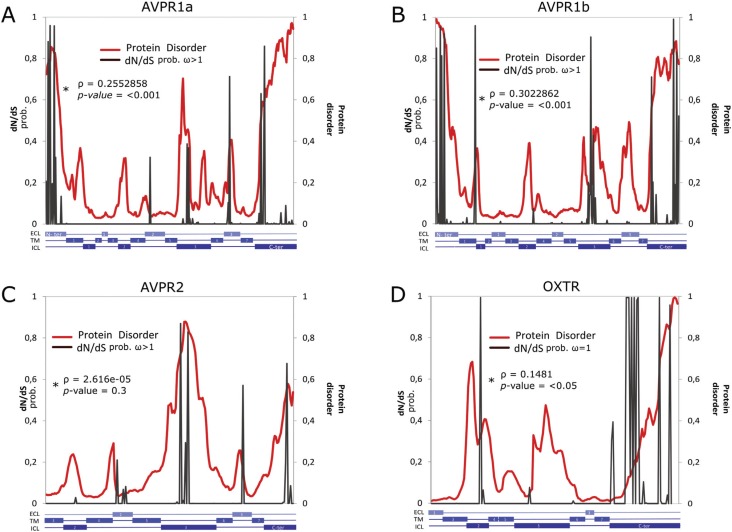
Bayes Empirical Bayes analyses. The probability of ω > 1 (sites under positive selection and/or relaxed constraint; A-C) or probability of ω = 1 (sites under neutrality and/or relaxed purifying selection; D) is shown in gray. Disorder degree (red) estimated for each residue of *Homo sapiens* AVPR1a (A), AVPR1b (B), AVPR2 (C), and OXTR (D). ECL: Extracellular; TM: Transmembrane; ICL: Intracellular. Patterns similar to those of other mammalian species were obtained (Table 3). ω = nonsynonymous/synonymous rate ratio. *Rho= Correlation between disorder value and the probability of being under positive selection or relaxed constraint.

Parameter estimations and log-likelihood values under models of variable ω indicated that the *OXTR* gene exhibited evolutionary constraint in placental mammals (Table 1; Table S2). The neutral model M1a, which assumes purifying and neutral ω values, explains the molecular evolution of *OXTR*, since the LRT is not significant for models that admit positive selection. In Table 1, it is possible to see that around 94% (p_0_=0.93893) of the OXTR sites are under purifying selection (ω_0_=0.03039) and the remaining 6% (p_1_=0.06107) are under neutrality (ω_1_=1). On the other hand, the same test indicated that the model M8, which admits positive selection, is the best-fit model for the molecular evolution of the AVP receptors regarding placental mammals. In other words, 4%, 3%, and 3% of the AVPR1a, AVPR1b, and AVPR2 sites, respectively, were suggested to be under positive selection or relaxed functional constraints (Table 1), whereas the remaining sites were suggested to be under purifying selection. These results were confirmed through Bayes Empirical Bayes analysis, one example being at position 404 in AVPR1b, shown to have 99% of probability of being under positive selection (Figure 2B). Notably, a glutamine at this position is fixed in all primates except for the Bushbaby (*Otolemur garnettii*; Table S3, Figure S1).

**Table 1:** Estimated parameters under different codon substitution models for *OXTR, AVPR1a*, *AVPR1b,* and *AVPR2*.

	Model	*dN/dS*	Estimated parameters	ℓ	*p value*
AVPR1a	M7: β	0.1504	[p=0.22139, q=1.22306]	-10046.63	M7 *vs* M8 *p* < **0.001**
	M8: β&ω	0.1399	p_0_=0.95125, (p_2_ =0.04875), ω_2_=1.04782	-10036.96	
AVPR1b	M7: β	0.1723	[p=0.33171, q=1.56124]	-10988.96	M7 *vs* M8 *p* < **0.001**
	M8: β&ω	0.1709	p_0_=0.96686, (p_2_ =0.03314), ω_2_ =1.39536	-10973.98	
AVPR2	M7: β	0.1123	[p=0.17525, q=1.33373]	-5496.23	M7 *vs* M8 *p* = **0.04**
	M8: β&ω	0.1053	p_0_=0.96649, (p_2_ =0.03351), ω_2_ =1.00000	-5493.606	
OXTR	M1a: Nearly	0.0896	p_0_=0.93893, (p_1_=0.06107)	-4401.07	
	Neutral				
			(ω_0_=0.03039), (ω_1_=1.00000)		M1a *vs* M2a *p* > 0.999
	M2a: Selection	0.0896	p_0_=0.93893, p_1_=0.06107, (p_2_=0.00000)	-4401.07	
			(ω_0_=0.03039), (ω_1_=1.00000), ω_2_=36.81273		

p0 = proportion of sites where ω < 1, p1 = proportion of sites where ω = 1, and p2 = proportion of sites where ω > 1 (selection models only); ω0 < 1 (negative selection), ω1 ≅ 1 (neutral or relaxing selection), and ω2 > 1 (positive selection). ℓ= Log likelihood values. Likelihood ratio tests were performed between neutral models (M1a- nearly neutral, and M7 - beta) and models that identify positive selection (M2a - selection, and M8, β&ω - beta +selection). The comparisons M1 vs M2 and M7 vs M8 had 2 degrees of freedom. Within parentheses: fixed parameters; within brackets: βparameters p and q. dN/dS = non-synonymous/synonymous rate ratio.

No difference in the evolutionary patterns was obtained by considering the data with or without gaps (see Materials and Methods, Data analysis section). In summary, *OXTR* seems to be under evolutionary constraint in placental mammals, whereas *AVPR1a, AVPR1b*, and *AVPR2* exhibit higher evolutionary rates, suggesting they are underrelaxed functional constraints or are experiencing positive selection.

### OXTR, AVPR1a, AVPR1b, and AVPR2 structures as determined through their intrinsic protein disorder patterns

**Figure 3:**
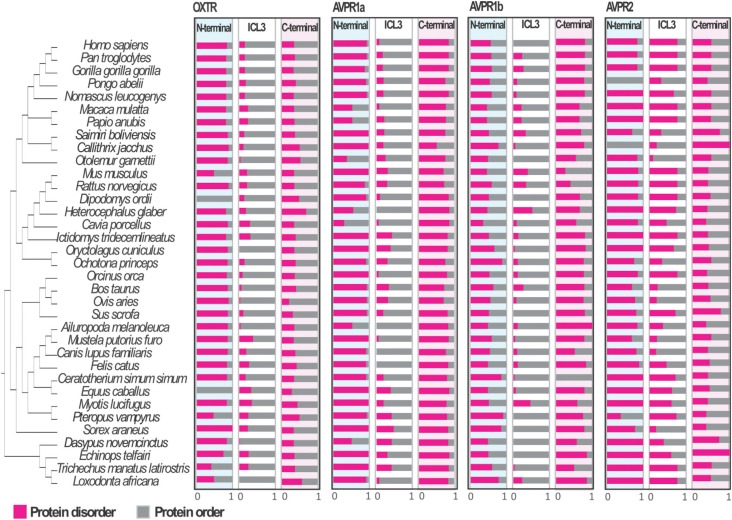
Protein disorder content for N-terminal, ICL3, and C-terminal regions between paralogues and among orthologues of OXTR, AVPR1a, AVPR1b, and AVPR2.

Disorder content was observed within N-terminal, ICL3, and C-terminal regions in all receptors (Tables S4 – S7). However, the proportions were significantly different (Table 2): specifically, the AVPR1a, AVPR2, and OXTR N-terminal regions and the AVPR1a C-terminus showed the highest proportions of residues predicted as being disordered (> 82%) across the placental mammalian species studied here. In the ICL3 region, the highest disorder content (57%) was found for AVPR2 (Table 2; Figure 3). As indicated in the Table 2, all values were significantly different (*p* < 0.001), when comparisons were made considering the same region of AVPR1a, AVPR1b, AVPR2 and OXTR. Pairwise comparisons of the regions in each receptor also showed significant differences, with the exception of AVPR2 C-terminal *vs* AVPR2 ICL3 (0.5209 *vs* 0.56990; *p* =0.3202).

**Table 2:** Median values of the proportions of residues predicted as intrinsically disordered for the N-terminal, ICL3, and C-terminal regions of OXTR, AVPR1a, AVPR1b, and AVPR2, as well as comparison among receptors for each domain considering 35 placental mammals^a^.

	N-terminal	ICL3	C-terminal
	Median	Median	Median
**AVPR1a**	0.8848 (0.32075–1)	0.2584 (0.061224–0.509091)	0.8262 (0.525424–0.898305)
**AVPR1b**	0.5995 (0.361111–0.944444)	0.2090 (0.037037–0.555556)	0.7334 (0.268293–0.883117)
**AVPR2**	0.8885 (0.394737–1)	0.5699 (0.102564–0.811295)	0.5209 (0.380952–1)
**OXTR**	0.8345 (0.425–1)	0.1962 (0.022727–0.403846)	0.3978 (0.210526–0.706897)
*p* (among paralogous regions)	< 0.001	< 0.001	< 0.001

a Median values and Kruskall Wallis tests with *p* values comparing the mean rank of intrinsic protein disorder content. The last line presents the values when the same region is compared in each protein. Pairwise comparisons between regions of each receptor also showed significant results, with the exception of the AVPR2 C-terminal *vs* AVPR2 ICL3 (*p* =0.3202).

To test whether the orders differed regarding their disorder content, a Kruskal-Wallis test was performed. Except for the C-terminal region of AVPR2, none of the analyzed domains differed among orders, suggesting that the disorder content is homogeneous for each orthologue (data not shown, *p* value > 0.05). The AVPR2 protein suggested difference among orders considering disorder content in the C-terminal region (*p*=0.046). Pairwise comparisons showed that Primates (median 0.5238) differed from Rodentia (median 0.4286), Carnivora (median 0.4524), and Chiroptera (median 0.4066). However, this significance did not occur when multiple tests adjustment was performed (Table S8). Our analyses also showed that the intrinsic disorder content differed more between paralogues than among orthologues (Table 3; Figure 3). The pairwise results indicated that the N-terminal regions of the paralogues AVPR1a and AVPR2 were similar, while that predicted for AVPR1b differed significantly. For ICL3, only AVPR2 had a different disorder degree content in comparison with the others, whereas the C-terminal regions of all the receptors were statistically different from each other (Table 3). Thus, based on the protein intrinsic disorder content, the results just partially replicate the phylogenetic pattern of the paralogues suggested by Lagman *et al.*, (2013) and Yamaguchi *et al.*, (2012). Additionally, our analyses revealed a positive correlation between disorder value and the probability of beingunder positive selection or relaxed constraint for all receptors (Figure 2).

**Table 3:** Mann-Whitney test results for pairwise comparisons* between N-terminal, ICL3, and C-terminal regions of OXTR, AVPR1a, AVPR1b, and AVPR2 regarding their intrinsic disorder degree content.

N-terminal		AVPR1a	AVPR1b	AVPR2	OXTR
	AVPR1a		0.000	0.402	0.000
	AVPR1b			0.000	0.000
	AVPR2				0.182
	OXTR				
ICL3		AVPR1a	AVPR1b	AVPR2	OXTR
	AVPR1a		0.149	0.000	0.610
	AVPR1b			0.000	0.654
	AVPR2				0.000
	OXTR				
C-terminal		AVPR1a	AVPR1b	AVPR2	OXTR
	AVPR1a		0.001	0.000	0.000
	AVPR1b			0.000	0.000
	AVPR2				0.000
	OXTR				

* *p* values after Bonferroni corrections.

### AVPR1a, AVPR1b, and AVPR2 and their SLiMs

Signals of positive selection or relaxed constraint were detected in *AVPR1a, AVPR1b*, and *AVPR2*, indicating that some changes highlighted here could have implications for adaptive novelties in placental mammals. Therefore, we considered the sites located at IDRs and with a high probability (> 65%) of being under positive selection or relaxed functional constraint as being the most relevant to explore in AVPR1a, AVPR1b, and AVPR2 for the possible presence of SLiMs (Tables S9, S3, and S10, respectively).

Our results revealed that sites with a higher probability of being under positive selection or relaxed constraint differed among paralogues; *i.e*., no site under this condition was the same between AVPR1a, AVPR1b, and AVPR2. Thus, similar to what was seen to occur with receptor disorder contents, the paralogues differed more than the orthologues (Tables S9, S3, and S10). The higher amino acid change ratios among the AVPR1a, AVPR1b, and AVPR2 orthologues were apparently responsible for the gain and loss of SLiMs (Tables S1, S3, and S10–S11).

Illustrative examples of the gain or loss of SLiMs can be seen at certain positions in AVPR1a [37 and 43] (Table S3) and AVPR1b [8, 62, and 404] (Table S9), which present a high probability (> 95%) of being under positive selection or relaxed functional constraint. These sites are very diverse among the species with respect to their amino acid residues. For example, it is possible to observe that the predicted SLiM MOD_ProDKin_1, which phosphorylates the substrates of MAP kinases, contains 7 residues, and exhibits in its extremity positions (37 and 43) a high level of amino acid diversity across mammalian species (Table S11).

Position 404 of AVPR1b (C-terminal) has the highest statistical probability of being under positive selection (> 99%) and exhibits a high variability of amino acids with at least one moderately radical change (*i.e.* Glutamine > Isoleucine, GS 134 in Bushbaby); however, no SLiM was predicted at this site. Thus, unlike other cases described here, the putative taxa-specific roles promoted by these different residues were not connected with SLiMs. As mentioned, AVPR1b mediates important processes such as stress control through adrenocorticotropic hormone release in the hypothalamic-pituitary-adrenal axis. It is possible that other structural and functional conditions not tested or predicted here are responsible for the signal of positive selection in this AVPR1b region.

AVPR2, on the other hand, shows the largest number of differences among the mammalian species. For instance, humans, apes, and other old world monkeys present an Arginine at position 249 (ICL3; Table S10). Our predicted SLiM analysis showed that this residue creates two cleavage motifs in different protein sequences from the species (CLV_NRD_NRD_1 and CLV_PCSK_FUR_1), probably in combination with other surrounding amino acids. Additionally, a motif connected with a di-Arginine retention/retrieving signal is also observed (Table S10). This last motif functions as a quality control mechanism for correct folding and protein complex assembly (Table S10). Within NWms, squirrel monkey (*Saimiri boliviensis*) and marmoset (*Callithrix jacchus*) present a Proline at the same position (predicted as a moderately radical change by the GS), which can be related with cleavage by only one protein category (endopeptidases). The presence of an Arginine at position 249 in other non-primate mammals; *i.e*., naked mole rat (*Heterocephalus glaber*), guinea pig (*Cavia porcellus*), squirrel (*Ictidomys tridecemlineatus*), microbat (*Myotis lucifugus*), and armadillo (*Dasypus novemcinctus*) concomitant with other SLiM scenarios reveals that surrounding residues are also responsible for protein stretch recognition.

Another example involving primates and AVPR2 (ICL 3) can be found at position 257 (Table S10). Humans and apes present a Glycine at this position, which is connected with a predicted binding site for Tumor Necrosis Factor Receptor - Associated Factors (TRAF6) and a phosphorylation site for protein casein kinase-2. Macaque (*Macaca mulatta*) has a Serine at the same position (a moderately conservative change), and the presence of a Serine at this position is involved in GPCR heterodimerization through the SLiM LIG_14-3-3_3. Baboon (*Papio anubis*) also contains a Glycine at this position, but neither motif was detected, which is probably connected with changes in surrounding positions. Interestingly, the loss of the two motifs in the AVPR2 proteins of both macaque and baboon, which are phylogenetically closely related (Figure S1), probably preserved their functional identity; nevertheless, they have different amino acids at the AVPR2 257 position. Another interesting change involving AVPR2 is an Arginine at position 345 of the kangaroo rat species (*Dipodomys ordii;* Table S10). This species is the unique placental mammal studied here that has a predicted SLiM (DOC_MAPK_1), which mediates docking with Mitogen-Activated Protein Kinases (MAPKs). This functional SLiM has been recognized through experimental data (Reményi *et al.*, 2005). MAPKs are involved with cellular responses to a diverse array of stimuli including osmotic stress (Pearson *et al.*, 2001). Since AVPR2 promotes water homeostasis (Koshimizu *et al.*, 2012) and kangaroo rats live in limited water supply environments (Marra *et al.*, 2012), it is possible to suggest that this change has probable adaptive implications.

## Discussion

Orthologous and paralogous genes emerge from speciation and duplication (genomic and partial), respectively (Gabaldón and Koonin, 2013). As result, these two types of homologous genes can retain similar or identical functions. However, along the evolutionary timeline they can also diverge extensively with respect to their spheres of action (Neduva and Russell, 2005; Huang and Sarai, 2012).

Our findings show that orthologues of the oxytocin and vasopressin receptor family are somewhat more similar than paralogues with respect to their disorder content and the presence of sites with a high probability of being under positive selection or relaxed functional constraint. This is an expected pattern considering the processes responsible for emergence of these two types of homologs; *i.e*., speciation or duplication, respectively (Gabaldón and Koonin, 2013).

Our analyses also revealed that *OXTR* is under evolutionary constraint in placental mammals. However, this general pattern does not appear to have prevented branch-specific particularities such as described recently by our research group (Vargas-Pinilla *et al.*, 2015). In that study, we found signals of positive selection and that some OXT-OXTR forms are coevolved, probably influencing the emergence of adaptive novelties such as male parental care in some NWm species.

On the other hand, the best-fit model for molecular evolution of the placental mammalian *AVPR1a, AVPR1b*, and *AVPR2* genes admits sites with positive selection or relaxed functional constraint (Table 1). These results were based on alignments including sequences selected in accordance with the best coverage, and without considering sequence gaps. The particularities observed in the evolutionary patterns of these genes are probably connected with the wide spectrum of functions mediated by these receptors, which includes complex behaviors (*e.g* parental care, pair bonding, stress control, etc). As previously mentioned, this interconnected system of receptors is present throughout the animal kingdom, and their typical roles in placental mammals are likely exaptations of ancient function (Oumi *et al.*, 1996; Fujino *et al.*, 1999). The positive selection signal observed here in *AVPR1a, AVPR1b*, and *AVPR2* for placental mammals, as well as for *OXTR* in Primates (Vargas-Pinilla *et al.*, 2015) might represent the marks of evolutionary adaptations that these animals experienced. Additional evidence that the system is not evolving neutrally is provided by the incongruity found between the phylogenetic tree topology based on orthologues and that based on 35 species.

To explore this possibility further, we evaluated whether the identified structural changes might have functional implications. For instance, the pattern of protein disorder content of OXTR, AVPR1a, AVPR1b, and AVPR2 is greater in the N-terminus, ICL3, and C-terminus, which are important regions for interactions with other molecular elements (Jaakola *et al.*, 2005; Tovo-Rodrigues *et al.*, 2014). This arrangement of protein disorder content was positively correlated with higher rates of ω values (including ω=1 and ω > 1, Figure 2), showing that OXTR, AVPR1a, AVPR1b, and AVPR2 are functionally evolving to increase possibilities for protein interaction. In a related manner, Huang and Sarai (2012), using genomic data, suggested that the more recently emerged IDRs (present in putatively more derived mammalian lineages) have significantly higher evolutionary rates than ancient IDRs, although the reasons for this finding have not been explored by them.

Within the IDRs were found the great part of the sites with the highest probability of being under positive selection and/or or relaxed constraint, as indicated by BEB analysis, reinforcing the role of IDRs in the evolvability of this genetic system. Positive selection has already been reported for other GPCRs (Kosiol *et al.*, 2008), but to the best of our knowledge this is the first time that a signal of this nature has been connected with IDRs in a GPCR family.

In this study, SLiM prediction was only performed on sites located at IDRs and carrying a high probability of being under positive selection or relaxed functional constraint. This analysis was conducted to evaluate whether the identified amino acid changes might have functional implications. We presented several illustrative examples such as the presence of the SLiM DOC_MAPK_1 in the kangaroo rat AVPR2, which mediates docking with a kinase involved with cellular response to a diverse array of stimuli including osmotic stress (Pearson *et al.*, 2001). This unique characteristic among placental mammals might have adaptive implications, since the importance of AVPR2 in the hypothalamic-renal regulation of water and electrolyte homeostasis became evident when mutations in the *AVPR2* gene were associated with nephrogenic diabetes insipidus in humans, mice, dogs, and horses, a disease characterized by polyuria and polydipsia (Luzius *et al.*, 1992; Böselt *et al.*, 2009).

Taken together, our results indicate that these receptors have been subjected to distinct evolutionary forces during placental mammal evolution, generating unique protein disorder patterns as well as specific SLiMs, described subsequently as "evolvability pathways." Previous studies with other orthologous and paralogous genes have revealed similar tendencies. For example, a single Tryptophan > Cysteine amino acid change eliminates a C-mannosylation site in an Interleukin-12 rodent protein; this change is found in orthologues of other mammals. Furthermore, among two closely paralogous proteins of mouse (actin-like 6B; ACTL6B), only one contains the motif for binding C-terminal binding protein (Neduva and Russell, 2005).

Our results as a whole show also that the evolutionary pathways of the genes *OXTR, AVPR1a*, *AVPR1b*, and *AVPR2* combine both conserved features (*e.g*., the largest disorder content in the N-terminal, ICL3, and C-terminal domains is retained in both paralogues and orthologues) and evolutionary changes (*e.g.,* different linear motif scenarios between paralogues and orthologues). Whereas conserved molecular features are likely associated with similar/overlapping functions of receptors, changes can be related to innovation and specialization.

Regarding protein disorder levels, these receptors follow a similar trend as observed for the ligand OXT and AVP nonapeptides, which have been described as intrinsically disordered (Yedvabny *et al.*, 2015). The suggestion that the level of disorder is maintained throughout evolution to promote a wide range of interaction among proteins and other molecules has been subject of intense debate (Schlessinger *et al.*, 2011; Xue *et al.*, 2013). Here we described for the first time that the OXTR, AVPR1a, AVPR1b, and AVPR2 mammalian orthologues have relatively preserved disorder content and interaction motifs, whereas these are diverse among the paralogues (Figure 3).

Finally, our results improve the current knowledge of evolutionary forces within the mammalian lineage affecting a key neuroendocrine system. In addition, these findings provide the basis for future computational, *in vitro*, and *in vivo* functional studies that eventually might corroborate the hypotheses suggested here that particular taxa-specific changes in OXTR, AVPR1a, AVPR1b, and AVPR2 receptors had (have) implications for adaptations of placental mammals.

## Supplementary Material

The following online material is available for this article:

Table S1 - Species included in the analyses.

Table S2 - Estimated parameters under different codon substitution models.

Table S3 - Amino acid changes at AVPR1b positions and probability of being under positive selection and/or having relaxed functional constraints.

Table S4 - Disorder content within OXTR domains.

Table S5 - Disorder content within AVPR1a domains.

Table S6 - Disorder content within AVPR1b domains

Table S7 - Disorder content within AVPR2 domains

Table S8 - Mann-Whitney test results for AVPR2 C-terminal regions with regard to their intrinsic disorder degree content.

Table S9 - Amino acid changes at the AVPR1a positions and probability of being under positive selection and/or having relaxed functional constraints.

Table S10 - Amino acid changes at the AVPR2 positions positionsand probability of being under positive selection and/or having relaxed functional constraints.

Table S11 - Short linear interaction motifs (SLiMs) predicted for AVPR1a, AVPR1b, and AVPR2.

Figure S1 - Phylogenetic tree topology used in the analysis of molecular evolution.
