# Supplementary material for: Oxytocin and arginine vasopressin receptor evolution: implications for adaptive novelties in placental mammals
Source: Genet Mol Biol. 2016 Aug 8;39(4):646–57. doi: 10.1590/1678-4685-GMB-2015-0323 (PMC5127151; doi:10.1590/1678-4685-GMB-2015-0323)
Supplement: Supplementary file 1 [file 1415-4757-gmb-1678-4685-GMB-2015-0323-Suppl01.pdf]

Table S1

Species included in the molecular evolution, protein disorder content, and SLiM prediction analyses.

COMMON NAME	SPECIES	CHROMOSOME LOCATION			
		OXTR	***AVPR1a***	***AVPR1b***	***AVPR2***
Armadillo	*Dasypus novemcinctus*	JH570036:784,101-786,550 [c]	JH565922:556,112-559,574 [b]	JH570036:784,017-786,655 [c]	JH582165:726,593-726,983 [d]
Baboon	*Papio anubis*	2:51,424,043-51,438,993 [c]	11:58,804,281-58,808,842 [c]	1:157,797,540-157,803,573 [c]	X:141,377,448-141,379,013 [c]
Bushbaby	*Otolemur garnettii*	GL873534.1:2342065-2358892:1 [a]	GL873657.1:1899023-1902474:-1 [a]	GL873574.1:3114139-3119132:1 [a]	GL873768.1:708867-710781:1 [a]
Cat	*Felis catus*	A2:49379828-49397678:-1 [a]	B4:91171687-91175144:-1 [a]	F1:43714510-43720039:-1 [a]	X:124716115-124717412:1 [a]
Chimpanzee	*Pan troglodytes*	3:8934843-8953915:-1 [a]	12:26138951-26146969:1 [a]	1:185210857-185218136:-1 [a]	X:154592309-154594400:1 [a]
Cow	*Bos taurus*	22:17817626-17827292:1 [a]	5:50592290-50595766:1 [a]	16:3726354-3731874:-1 [a]	X:40036423-40038823:1 [a]
Dog	*Canis lupus familiaris*	20:9358197-9378248:1 [a]	10:6264789-6269662:-1 [a]	38:2440633-2446082:-1 [a]	X:121752780-121754771:1 [a]
Elephant	*Loxodonta africana*	scaffold_12:38254308-38286930:1 [a]	scaffold_2:52880385-52883832:-1 [a]	scaffold_13:11192320-11197748:-1 [a]	scaffold_120:2147361-2148562:-1 [a]
Ferret	*Mustela putorius furo*	GL896899.1:31840028-31948723:-1 [a]	GL897020.1:3747775-3751757:1 [a]	GL896951.1:8943499-8948417:-1 [a]	GL897267.1:201524-203187:1 [a]
Gibbon	*Nomascus leucogenys*	GL397298.1:16339634-16360389:-1 [a]	GL397261.1:49510312-49520425:1 [a]	GL397352.1:94927-102313:-1 [a]	GL397458.1:518385-520482:1 [a]
Gorilla	*Gorilla gorilla gorilla*	3:9043790-9060305:-1 [a]	12:61405397-61409230:-1 [a]	1:186190900-186198207:1 [a]	X:151892588-151894682:1 [a]
Guinea Pig	*Cavia porcellus*	scaffold_87:1912214-1924192:1 [a]	scaffold_9:35043412-35046721:1 [a]	scaffold_12:16779491-16788717:1 [a]	scaffold_148:1815080-1816468:-1 [a]
Horse	*Equus caballus*	16:7978752-7994543:1 [a]	6:79470329-79473986:-1 [a]	5:2416813-2424080:-1 [a]	X:122356320-122357636:1 [a]
Human	*Homo sapiens*	3: 8,792,094-8,811,314 [a]	12: 63,539,014-63,544,722 [a]	1: 206,223,976-206,231,639 [a]	X: 153,167,985-153,172,620 [a]
Kangaroo rat	*Dipodomys ordii*	scaffold_17231:11075-22474:1 [a]	scaffold_22850:14404-17904:-1 [a]	scaffold_14784:13772-19597:-1 [a]	scaffold_2502:8231-9676:1 [a]
Lesser hedgehog tenrec	*Echinops telfairi*	scaffold_7583: 30,612-34,235 [a]	JH980300:69,823,458-69,827,381 [c]	JH980299:32,534,576-32,540,588 [c]	JH980299:32,534,593-32,540,501 [c]
Macaque	*Macaca mulatta*	2:52233185-52248347:1 [a]	11:60385085-60388662:-1 [a]	1:164231937-164238892:1 [a]	X:152025855-152027027:1 [a]
Manatee	*Trichechus manatus latirostris*	JH594607:8,611,837-8,643,861 [c]	JH594692:9,606,138-9,610,156 [c]	JH594641:9,296,834-9,302,481 [d]	JH594692:9,606,713-9,607,150 [c]
Marmoset	*Callithrix jacchus*	15: 60,672,911-60,688,438 [a]	9:52,395,838-52,399,124 [c]	19:28,142,077-28,149,505 [c]	X: 140,442,277-140,443,205 [a]
Megabat	*Pteropus vampyrus*	scaffold_3394:60991-76592:-1 [a]	scaffold_5495:44926-48380:-1 [a]	scaffold_3164:7745-9572:1 [a]	scaffold_3867:45661-47320:1 [a]
Microbat	*Myotis lucifugus*	GL429775:16113316-16130498:1 [a]	GL429956:91339-95204:-1 [a]	GL429912:1435434-1437638:-1 [a]	GL429807:619705-621296:-1 [a]
Mouse	*Mus musculus*	6:112473684-112489808:-1 [a]	10:122448499-122453453:1 [a]	1:131599239-131612000:1 [a]	X:73892102-73895502:1 [a]
Naked mole-rat	*Heterocephalus glaber*	JH602044:3,370,683-3,384,804 [c]	JH602115:4,894,523-4,897,919 [c]	JH602120:5,237,036-5,241,760 [c]	JH602259:541,899-543,032 [c]
Orangutan	*Pongo abelii*	3: 61,221,011-61,236,340 [a]	12:63,230,436-63,233,748 [c]	1: 44,067,955-44,074,792 [a]	1:44,068,211-44,074,543 [d] *
Orca	*Orcinus orca*	XM_004274790.1 [c]	XM_004276585.1 [c]	XM_004282434.1 [c]	XM_004286432.1 [c]
Panda	*Ailuropoda melanoleuca*	GL192752.1:988763-1005431:-1 [a]	GL192683.1:339899-343408:1 [a]	GL193859.1:174036-178969:1 [a]	GL193934.1:263280-264996:-1 [a]
Pig	*Sus scrofa*	13:72419130-72434787:-1 [a]	5:30643445-30647686:-1 [a]	9:73020218-73023991:-1 [a]	X:142203295-142205695:1 [a]
Pika	*Ochotona princeps*	scaffold_368: 202,694-214,098 [a]	scaffold_15380:45,643-45,937 [c]	scaffold_198:4,918-8,501 [c]	scaffold_198:5,153-8,297 [c]
Rabbit	*Oryctolagus cuniculus*	GL018703:223681-234789:1 [a]	4:42141558-42149565:-1 [a]	16:66192701-66196755:1 [a]	GL018816:535306-537985:-1 [a]
Rat	*Rattus norvegicus*	4:207703615-207716373:-1 [a]	7:67528203-67532266:-1 [a]	13:53443235-53453450:1 [a]	1:152637478-152639106:-1 [a]
Rhinoceros	*Ceratotherium simum simum*	JH767842:234,877-251,435 [a]	JH767750:20,542,259-20,546,534 [c]	JH767737:31,932,525-31,937,567 [c]	JH767842:235,003-235,413 [c]
Sheep	*Ovis aries*	19:17656099-17664613:1 [a]	3:156234256-156237118:1 [a]	12:3,157,164-3,162,772 [c]	JH921656.1:16006-17521:1 [a]
Shrew	*Sorex araneus*	scaffold_153104:4,229-4,310 [c]	scaffold_247307:186-1,060 [c]	scaffold_233098:39,285-40,192 [c]	scaffold_2713: 5-1,189 [a]
Squirrel	*Ictidomys tridecemlineatus*	JH393412.1:4242983-4258110:1 [a]	JH393355.1:1790988-1795450:-1 [a]	JH393367.1:6416062-6421547:1 [a]	JH393619.1:788990-790674:1 [a]
Squirrel monkey	*Saimiri boliviensis boliviensis*	JH378125:19,963,441-19,977,143 [c]	JH378123:6,306,524-6,310,202 [c]	JH378142:19,163,569-19,170,664 [c]	JH378125:19,963,567-19,976,990 [c]

Database: [a]Ensemble; [b]UCSC; [c]NCBI; [d]Uniprot; *_Pongo pygmaeus_.
