# Supplementary material for: Oxytocin and arginine vasopressin receptor evolution: implications for adaptive novelties in placental mammals
Source: Genet Mol Biol. 2016 Aug 8;39(4):646–57. doi: 10.1590/1678-4685-GMB-2015-0323 (PMC5127151; doi:10.1590/1678-4685-GMB-2015-0323)
Supplement: Supplementary file 2 [file 1415-4757-gmb-1678-4685-GMB-2015-0323-Suppl12.pdf]

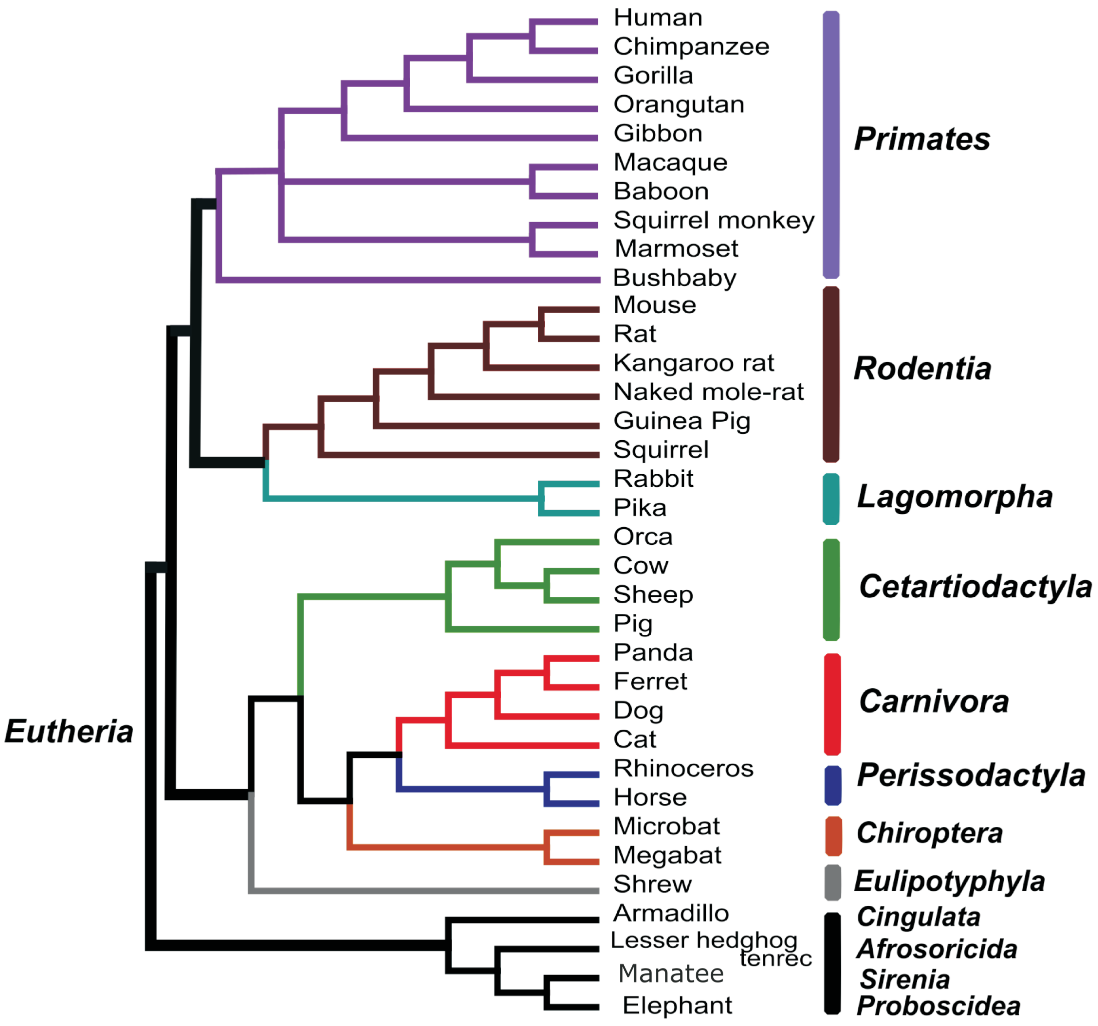

Figure S1 - Phylogenetic tree topology used in the analysis of molecular evolution. The analysis included 35 placental mammalian species (as described in the Materials and Methods section).
