# Supplementary material for: Oxytocin and arginine vasopressin receptor evolution: implications for adaptive novelties in placental mammals
Source: Genet Mol Biol. 2016 Aug 8;39(4):646–57. doi: 10.1590/1678-4685-GMB-2015-0323 (PMC5127151; doi:10.1590/1678-4685-GMB-2015-0323)
Supplement: Supplementary file 3 [file 1415-4757-gmb-1678-4685-GMB-2015-0323-Suppl02.pdf]

Table S2

Estimated parameters under different codon substitution models for *OXTR*, *AVPR1a*, *AVPR1b*, and *AVPR2*.

	Model	*dN/dS*	Estimated parameters	ℓ	*p value*
AVPR1a	M1a: Nearly Neutral	0.2000	p_0=0.84972, (p_1=0.15028)	-10155.10	
			(ω_0=0.05852), (ω_1=1.00000)		>0.999
	M2a: Selection	0.2000	p_0=0.84972, p_1=0.12155, (p_2=0.02873)	-10155.10	
			(ω_0=0.05852), (ω_1=1.00000), ω_2=1.00000		
AVPR1b	M1a: Nearly Neutral	0.2104	p_0=0.85767, (p_1=0.14233)	-11068.99	
			(ω_0=0.07939), (ω_1=1.00000)		0.4
	M2a: Selection	0.1247	p_0=0.85731, p_1=0.13855, (p_2=0.00414)	-11068.11	
			(ω_0=0.07985), (ω_1=1.00000), ω_2=2.65042		
AVPR2	M1a: Nearly Neutral	0.1678	p_0=0.86382, (p_1=0.13618)	-5563.60	
			(ω_0=0.03663), (ω_1=1.00000)		>0.999
	M2a: Selection	0.1678	p_0=0.86381, p_1=0.00588, (p_2=0.13030)	-5563.60	
			(ω_0=0.03663), (ω_1=1.00000), ω_2=1.00000		
OXTR	M7: β	0.0617	[p=0.17515, q=2.45228]	-4316.14	>0.999
			p_0=0.99688,		
	M8: β & ω	0.0620	(p_2 =0.00312), ω_2=1.00000	-4316.09	

p0 = proportion of sites where ω < 1, p1 = proportion of sites where ω = 1, and p2 = proportion of sites where ω > 1 (selection models only); ω0 < 1 (negative selection), ω1 ≅ 1 (neutral or relaxing selection), and ω2 >1 (positive selection). ℓ= Log likelihood values. Likelihood ratio tests were performed between neutral models (M1a- nearly neutral, and M7 - beta) and models that identify positive selection (M2a - selection, and M8, β & ω - beta +selection). The comparisons M1 vs M2 and M7 vs M8 had 2 degrees of freedom. Within parentheses: fixed parameters; within brackets: $^{\beta}$parameters p and q. dN/dS = non-synonymous/synonymous rate ratio.
