# Supplementary material for: Oxytocin and arginine vasopressin receptor evolution: implications for adaptive novelties in placental mammals
Source: Genet Mol Biol. 2016 Aug 8;39(4):646–57. doi: 10.1590/1678-4685-GMB-2015-0323 (PMC5127151; doi:10.1590/1678-4685-GMB-2015-0323)
Supplement: Supplementary file 4 [file 1415-4757-gmb-1678-4685-GMB-2015-0323-Suppl03.pdf]

Table S3

Amino acid changes at the AVPR1b positions with a higher probability (> 65%) of being under positive selection and/or having relaxed functional constraints.

Protein region	Nucleotide	Amino acid	Grantham score	Mammalian species																																		
				Human	Chimpanzee	Gorilla	Orangutan	Gibbon	Macaque	Baboon	Squirrel monkey	Marmoset	Bushbaby	Mouse	Rat	Kangaroo rat	Naked mole rat	Guinea pig	Squirrel	Rabbit	Pika	Orca	Cow	Sheep	Pig	Panda	Ferret	Dog	Cat	Horse	Rhinoceros	Microbat	Megabat	Shrew	Manatee	Elephant	Lesser hedgehog tenrec	Armadillo
N-terminal	GAT->AAT	2Asp->Asn	23	0	0	0	0	0	0	0	0	0	0	0	1		1	0	0	0		0	0	0	0	0	0		0	0	0	0			0	0		0
	GAT->GAA	2Asp->Glu	45																														1					
	GAT->GAG	2Asp->Glu	45																																		1	
	GAT->ACT	2Asp->Thr	85																									1										
	GAT->GGC	2Asp->Gly	94													1																						
	GAT->GCT	2Asp->Ala	126																															1				
	GAT->GTT	2Asp->Val	152																		1																	
	GAT->AAT	8Asp->Asn +	23	0_j	0	0		0	0	0	1	1																										
	GAT->GAG	8Asp->Glu +	45				1																															
	GAT->ACT	8Asp->Thr +	85											1_{kj}	1_{kj}				1																			
	GAT->ACG	8Asp->Thr +	85																				1	1														
	GAT->GGT	8Asp->Gly +	94																		1_j																	
	GAT->GCT	8Asp->Ala +	126										1			1_k		1_k								1_j	1	1_{kj}	1_j	1	1	1		1	1	1	1	1
	GAT->GCA	8Asp->Ala +	126																														1					
	GAT->GCG	8Asp->Ala +	126														1																					
	GAT->GTT	8Asp->Val+	152																	1_j																		
	GAT->ATC	8Asp->Ile +	168																			1_j																
	GAT->TTG	8Asp->Leu +	172																						1_j													
	AAC->AGC	10Asn->Ser	46	0	0	0	0	0	0	0	0	0	1			0						1			0		0		0	0	0		0					0
	AAC->GAC	10Asn->Asp	23																													1_m						
	AAC->AGC	10Asn->Ser	46																							1_l												
	AAC->AGC	10Asn->Ser	46																									1_l										
	AAC->AGT	10Asn->Ser	46																	1	1																	
	AAC->CAG	10Asn->Gln	46																															1_l				
	AAC->TCC	10Asn->Ser	46																																1_l			
	AAC->AGC	10Asn->Ser	46																																		1_l	
	AAC->ACC	10Asn->Thr	65											1_l	1_l																							
	AAC->ACC	10Asn->Thr	65																																	1_l		
	AAC->GCT	10Asn->Ala	111														1						1_l	1_l														
	AAC->ATC	10Asn->Ile	133															1																				
	AAC->ATA	10Asn->Ile	149																1_l																			
	CGG->CAG	14Arg->Gln	43	0	0	0	0						1			0	1	1									1											
	CGG->GAA	14Arg->Glu	54																		1																	
	CGG->GAG	14Arg->Glu	54																																1			
	CGG->TGG	14Arg->Trp	101						1	1																				1								
	CGG->CTG	14Arg->Leu	102																							1												
	CGG->GGG	14Arg->Gly	125					1			1	1		1	1				1	1		1	1	1				1	1			1	1	1		1	1	1
	CGG->GGC	14Arg->Gly	125																												1							
	CGG->GGA	14Arg->Gly	125																						1													
ICL1	CTG->CAT	62Leu->His +	99	0	0	0	0	0	0	0	0		0		1			1												0	0							
	CTG->GCA	62Leu->Ala +	96																			1																
	CTG->CCG	62Leu->Pro +	98														1						1	1														
	CTG->CCA	62Leu->Pro +	98									1							1						1			1	1							1	1_i	
	CTG->CCC	62Leu->Pro +	98													1				1						1												
	CTG->CCT	62Leu->Pro +	98																		1														1			
	CTG->CGA	62Leu->Arg +	102																								1_i											
	CTG->CGG	62Leu->Arg +	102																														1_i	1_i				

	CTG->CAG	62Leu->Gln +	113											1																		1						
	CTG->TCA	62Leu->Ser +	145																																			1
ICL3	GGA->GCG	241Gly->Ala	60	0	0	0	0	0	0	0	0	0	0			1				0	0	0	0	0	0	0	0	0	0			0	0	0	0	0	0	1
	GGA->GCA	241Gly->Ala	60																1																			
	GGA->GAA	241Gly->Glu	98																												1							
	GGA->AGA	241Gly->Arg	125											1				1												1								
	GGA->AGG	241Gly->Arg	125												1		1																					
C-terminal	CAG->CGT	360Gln->Arg	43	0	0	0	0	0	0	0	0	0	1			0												0			0					0		
	CAG->CGG	360Gln->Arg	43														1	1	1	1	1					1	1		1_d	1		1			1			
	CAG->AAG	360Gln->Lys	53											1	1																							
	CAG->TCG	360Gln->Ser	68																															1_m				
	CAG->CCG	360Gln->Pro	76																																			1
	CAG->GGG	360Gln->Gly	87																			1	1	1	1								1				1	
	GAA->CAA	**404Glu->Gln +**	29	0	0	0	0	0	0	0	0	0								1		0			1	0	0	0	0	1	0	0	0		0	0	0	
	GAA->CAG	**404Glu->Gln +**	29																																			1
	GAA->AGA	**404Glu->Arg +**	54																					1														
	GAA->AAA	**404Glu->Lys +**	56																				1															
	GAA->ACG	**404Glu->Thr +**	65																															1				
	GAA->GGA	**404Glu->Gly +**	98																		1																	
	GAA->GCG	**404Glu->Ala +**	107											1			1																					
	GAA->GCA	**404Glu->Ala +**	107												1	1		1	1																			
	GAA->ATA	**404Glu->Ile +**	134										1																									
	TTG->ACA	410Leu->Thr	92	0	0	0	0	0	0	0	0	0		0	0	0	0	0	0					1											0	0		0
	TTG->GCA	410Leu->Ala	96																				1															
	TTG->CCA	410Leu->Pro	98																															1			1	
	TTG->GAA	410Leu->Glu	138																			1																
	TTG->TCG	410Leu->Ser	145										1								1				1	1												
	TTG->TCC	410Leu->Ser	145																	1																		
	TTG->TCA	410Leu->Ser	145																								1	1		1	1	1	1					
	TTG->TCT	410Leu->Ser	145																										1									

Sites identified in the Bayes Empirical Bayes (BEB) analysis with a probability higher than 65% of being under ω > 1, which indicates positive selection. + Positively selected sites (p >95%); bold text indicates positively selected sites (p >99%). AVPR1b regions: N-terminal= Amino-terminus; ICL 1= Intracellular 1; ICL 3 = Intracellular 3; and C-terminal = Carboxyl-terminus. Zero (0) indicates the amino acid present in the *Homo sapiens* reference sequence, whereas 1 indicates a variant amino acid.

The nature of the modification does not represent an ancestry and descendant relationship. Grantham scores are predicted as conservative (0–50), moderately conservative (51–100), moderately radical (101–150), or radical (>151). Subscripted letters indicate the predicted presence of specific Eukaryotic Linear Motifs (as depicted using the code shown in Table S11). Scientific names of the species can be found in Table S1.
