# Supplementary material for: Oxytocin and arginine vasopressin receptor evolution: implications for adaptive novelties in placental mammals
Source: Genet Mol Biol. 2016 Aug 8;39(4):646–57. doi: 10.1590/1678-4685-GMB-2015-0323 (PMC5127151; doi:10.1590/1678-4685-GMB-2015-0323)
Supplement: Supplementary file 5 [file 1415-4757-gmb-1678-4685-GMB-2015-0323-Suppl04.pdf]

Table S4

Disorder content* within each domain of OXTR in which intrinsic disorder was observed.

Species	N-terminal	ICL2	ICL3	ECL3	C-terminal
Human	0.88	0.26	0.16	-	0.36
Chimpanzee	0.85	0.28	0.16	-	0.38
Gorilla	0.85	0.28	0.16	-	0.34
Orangutan	0.85	-	0.19	-	0.41
Gibbon	0.85	0.26	0.16	-	0.34
Macaque	0.85	0.28	0.26	-	0.36
Baboon	0.85	0.28	0.26	-	0.37
Squirrel monkey	0.90	0.28	0.15	-	0.38
Marmoset	0.93	-	0.13	-	0.52
Bushbaby	0.90	0.25	0.04	-	0.54
Mouse	0.50	0.26	0.23	-	0.36
Rat	0.93	0.26	0.23	-	0.36
Kangaroo rat	-	-	0.15	-	0.50
Naked mole rat	0.85	0.37	0.20	-	0.71
Guinea pig	0.90	0.26	0.31	-	0.36
Squirrel	0.85	0.26	0.33	-	0.40
Rabbit	0.90	0.27	-	-	0.36
Pika	0.90	0.37	0.15	-	0.40
Orca	0.88	0.18	0.10	-	0.38
Cow	0.90	-	0.06	-	0.41
sheep	0.93	0.20	0.03	-	0.21
Pig	0.90	0.22	0.12	-	0.32
Panda	0.90	0.22	0.04	-	0.36
Ferret	0.90	0.20	0.40	-	0.34
Dog	0.90	0.21	0.20	-	0.39
Cat	0.88	0.21	0.29	-	0.43
Rhinoceros	0.88	-	0.18	-	0.36
Horse		-	0.35	-	0.29
Microbat	0.87	0.21	0.37	-	0.45
Megabat	0.49	-	0.27	-	0.51
Shrew	1.00	0.28	0.25	0.50	0.34
Armadillo	0.88	-	0.02	-	0.35
Lesser hedgehog tenrec	0.78	-	0.27	-	0.37
Manatee	0.43	0.21	0.27	-	0.38
Elephant	0.50	0.22	-	-	0.58

*Disorder content: Proportion of amino acids predicted as intrinsically disordered considering the total number of amino acid residues in each region. The domains that lack disorder content for all the species were not included in the table. The symbol “–“ indicates the absence of amino acid residues predicted as intrinsically disordered.
