# Supplementary material for: Oxytocin and arginine vasopressin receptor evolution: implications for adaptive novelties in placental mammals
Source: Genet Mol Biol. 2016 Aug 8;39(4):646–57. doi: 10.1590/1678-4685-GMB-2015-0323 (PMC5127151; doi:10.1590/1678-4685-GMB-2015-0323)
Supplement: Supplementary file 7 [file 1415-4757-gmb-1678-4685-GMB-2015-0323-Suppl08.pdf]

Table S8

Mann-Whitney test results for pairwise comparisons between mammalian orders of AVPR2 C-terminal regions with regard to their intrinsic disorder degree content.

Orders	Primates	Rodentia	Lagomorpha	Cetartiodactyla	Carnivora	Perissodactyla	Chiroptera	Eulipotyphyla	Cingulata	Afrosoricida	Sirenia	Proboscidea
Primates		0.004	0.338	0.185	0.05	0.067	0.039	0.085	0.301	0.12	0.301	0.168
		0.264*	22.308*	12.21*	3.3*	4.422*	2.574*	5.61*	19.866*	7.92*	19.87*	11.088*
Rodentia			0.094	0.282	0.588	0.495	0.495	0.203	0.13	0.13	0.13	0.13
			6.204*	18.612*	38.808*	32.67*	32.67*	13.398*	8.58*	8.58*	8.58*	8.58*
Lagomorpha				0.643	0.481	0.439	0.121	0.221	0.221	0.221	0.221	1
				42.438*	31.746*	28.974*	7.986*	14.586*	14.586*	14.586*	14.59*	1
Cetartiodactyla					0.564	0.643	0.165	0.157	0.48	0.157	0.48	0.48
					37.224*	42.438*	10.89*	10.362*	31.68*	10.362*	31.68*	31.68*
Carnivora						0.481	0.277	0.157	0.157	0.157	0.157	0.48
						31.746*	18.282*	10.362*	10.362*	10.362*	10.36*	31.68*
Perissodactyla							0.221	0.221	0.221	0.221	0.221	0.221
							14.586*	14.586*	14.586*	14.586*	14.59*	14.586*
Chiroptera								0.221	0.221	0.221	0.221	0.221
								14.586*	14.586*	14.586*	14.59*	14.586*
Eulipotyphyla									0.317	0.317	0.317	0.317
									20.922*	20.922*	20.92*	20.922*
Cingulata										0.317	0.317	0.317
										20.922*	20.92*	20.922*
Afrosoricida											0.317	0.317
											20.92*	20.922*
Sirenia												0.317
												20.922*
Proboscidea												

**p* values after Bonferroni corrections.
