# Supplementary material for: Oxytocin and arginine vasopressin receptor evolution: implications for adaptive novelties in placental mammals
Source: Genet Mol Biol. 2016 Aug 8;39(4):646–57. doi: 10.1590/1678-4685-GMB-2015-0323 (PMC5127151; doi:10.1590/1678-4685-GMB-2015-0323)
Supplement: Supplementary file 8 [file 1415-4757-gmb-1678-4685-GMB-2015-0323-Suppl09.pdf]

Table S9

Amino acid changes at the AVPR1a positions with a higher probability (> 65%) of being under positive selection and/or having relaxed functional constraints.

Protein region	Nucleotide	Amino acid	Grantham score	Mammalian species																																		
				Human	Chimpanzee	Gorilla	Orangutan	Gibbon	Macaque	Baboon	Squirrel monkey	Marmoset	Bushbaby	Mouse	Rat	Kangaroo rat	Naked mole rat	Guinea pig	Squirrel	Rabbit	Pika	Orca	Cow	Sheep	Pig	Panda	Ferret	Dog	Cat	Horse	Rhinoceros	Microbat	Megabat	Shrew	Manatee	Elephant	Lesser hedgehog tenrec	Armadillo
N-terminal	ACA->GCC	28 Thr->Ala	58	0	0	0	0	0	0	0	0	0	1									0	0	0	0					1	1	1	1	1				0
	ACA->GCG	28 Thr->Ala	58																									1										
	ACA->AGC	28 Thr->Ser	58											1							1																	
	ACA->GCT	28 Thr->Ala	58													1																						
	ACA->AGT	28 Thr->Ser	58															1																				
	ACA->GGC	28 Thr->Gly	59												1					1									1									
	ACA->GGT	28 Thr->Gly	59																							1												
	ACA->CAG	28 Thr->Glu	65																1																			
	ACA->AAT	28 Thr->Asn	65														1																					
	ACA->CGT	28 Thr->Arg	71																								1											
	ACA->ATG	28 Thr->Met	81																																	1		
	ACA->TTG	28 Thr->Leu	92																																		1	
	ACA->CTG	28 Thr->Leu	92																																1			
	GAG->GAC	37 Glu->Asp +	45	0	0	0	0	0_{g}	0	0	0_{n}	0		0_{m}	0_{m}		0	0		0			0	0		0_{m}		1	1_{m}		0	0	0	1	0_{n}	0	0_{no}	0_{m}
	GAG->AGA	37 Glu->Arg +	54																		1																	
	GAG->AAA	37 Glu->Lys +	56										1									1_{m}			1													
	GAG->AAG	37 Glu->Lys +	56																											1_{n}								
	GAG->GGA	37 Glu->Gly +	98													1											1_{m}											
	GAG->GGT	37 Glu->Gly +	98																1																			
	AGG->AAG	43 Arg->Lys +	26	0	0_{m}	0	1	0	0	0	1		0				0	0										0		0		0	0					
	AGG->AAA	43 Arg->Lys +	26									1																										
	AGG->CAG	43 Arg->Gln +	43																															1			1	
	AGG->CAA	43 Arg->Gln +	43																																			1_{m}
	AGG->GAG	43 Arg->Glu +	54													1																						
	AGG->CTG	43 Arg->Leu +	102																																	1		
	AGG->GCG	43 Arg->Ala +	112																		1	1_{m}	1	1														
	AGG->GCC	43 Arg->Ala +	112																						1													
	AGG->GGG	43 Arg->Gly +	125											1_{m}	1_{m}					1						1_{m}	1_{m}		1_{m}		1				1			
	AGG->GGA	43 Arg->Gly +	125																1_{m}																			
ECL3	GTC->ATC	321 Val->Ile	29	0	0	0	0	0	0	0	0	1		0	1	1	0	0	1	0	0					0	0	0	0		0						0_{o}	0
	GTC->GCC	321 Val->Ala	64																														1		1_{o}	1_{o}		
	GTC->CCC	321 Val->Pro	68																			1										1						
	GTC->CAC	321 Val->His	84																											1								
	GTC->AAG	321 Val->Lys	97										1																									
	GTC->GAG	321 Val->Glu	121																															1				
	GTC->TCC	321 Val->Ser	124																				1	1	1													
	GTC->AAC	321 Val->Asn	133																	1	1										1							
	AAA->ACA	372 Lys->Thr	78	0	0	0	1	0	0	0		0		0	0		1	0	0				1		1	1	1	1			1		1	0	0	0		0
	AAA->AGA	372 Lys->Arg	26								1		1																									
	AAA->CAA	372 Lys->Gln	53													1																					1	

AAA->ACG	372 Lys->Thr	78					1					1	1		
AAA->AAC	372 Lys->Asn	94	1	1											
AAA->ATA	372 Lys->Ile	102			1										
AAA->GCA	372 Lys->Ala	106													1

Sites identified in the Bayes Empirical Bayes (BEB) analysis with a probability higher than 65% of being under ω > 1, which indicates positive selection. + Positively selected sites (*p* >95%); bold text indicates positively selected sites (*p* >99%). AVPR1a regions: N-terminal= Amino-terminus; and ECL 3 = Extracellular 3. Zero (0) indicates the amino acid present in the *Homo sapiens* reference sequence, whereas 1 indicates a variant amino acid.

The nature of the modification does not represent an ancestry and descendant relationship. Grantham scores are predicted as conservative (0–50), moderately conservative (51–100), moderately radical (101–150), or radical (>151). Subscripted letters indicate the predicted presence of specific Eukaryotic Linear Motifs (as depicted using the code shown in Table S11). Scientific names of the species can be found in Table S1.
