# Supplementary material for: Oxytocin and arginine vasopressin receptor evolution: implications for adaptive novelties in placental mammals
Source: Genet Mol Biol. 2016 Aug 8;39(4):646–57. doi: 10.1590/1678-4685-GMB-2015-0323 (PMC5127151; doi:10.1590/1678-4685-GMB-2015-0323)
Supplement: Supplementary file 9 [file 1415-4757-gmb-1678-4685-GMB-2015-0323-Suppl10.pdf]

**Table S10**

Amino acid changes at the AVPR2 positions with a higher probability (> 65%) of being under positive selection and/or having relaxed functional constraints.

| Protein region | Nucleotide | Amino acid | Grantham score | Mammalian species | | | | | | | | | | | | | | | | | | | | | | | | | | | | | | | | | | |
|---|---|---|---|---|---|---|---|---|---|---|---|---|---|---|---|---|---|---|---|---|---|---|---|---|---|---|---|---|---|---|---|---|---|---|---|---|---|---|
| | | | | Human | Chimpanzee | Gorilla | Orangutan | Gibbon | Macaque | Baboon | Squirrel monkey | Marmoset | Bushbaby | Mouse | Rat | Kangaroo rat | Naked mole rat | Guinea pig | Squirrel | Rabbit | Pika | Orca | Cow | Sheep | Pig | Panda | Ferret | Dog | Cat | Horse | Rhinoceros | Microbat | Megabat | Shrew | Manatee | Elephant | Lesser hedgehog tenrec | Armadillo |
| ICL3 | AGG->GTA | 249 Arg->Val | 96 | $0_{abd}$ | $0_{abd}$ | $0_{abd}$ | $0_{abd}$ | $0_{abd}$ | $0_{abd}$ | $0_{abd}$ | | | | 0 | 0 | 0 | $0_a$ | $0_{ad}$ | $0_{bcd}$ | | $1_a$ | | 0 | 0 | 0 | | | | | | | $0_{ab}$ | | 0 | | | | $0_a$ |
| | AGG->CCG | 249 Arg->Pro | 103 | | | | | | | | $1_a$ | $1_a$ | | | | | | | | | | | | | | | | | | | | | | | | | | |
| | AGG->AGC | 249 Arg->Ser | 110 | | | | | | | | | | | | | | | | | | | | | | | | | | | | | | | | | $1_{abd}$ | | |
| | AGG->GGG | 249 Arg->Gly | 125 | | | | | | | | | | 1 | | | | | | | | | | | | | 1 | | | 1 | | | | | | | | | |
| | AGG->GGA | 249 Arg->Gly | 125 | | | | | | | | | | | | | | | | | $1_a$ | | $1_a$ | | | | 1 | 1 | 1 | | 1 | $1_a$ | | $1_a$ | | | | | |
| | AGG->GGC | 249 Arg->Gly | 125 | | | | | | | | | | | | | | | | | | | | | | | | | | | | | | | | $1_{ad}$ | | $1_a$ | |
| | GGT->AGT | 257 Gly->Ser | 56 | $0_{fg}$ | $0_{fg}$ | $0_{fg}$ | $0_{fg}$ | $0_{fg}$ | 1 | 0 | | | | | | $1_g$ | | | | | | | | | | $1_{eg}$ | | | $1_{eg}$ | | | 0 | 0 | | 1 | | | |
| | GGT->AGC | 257 Gly->Ser | 56 | | | | | | | | 1 | 1 | $1_g$ | $1_{efg}$ | $1_{efg}$ | | | | | 1 | | $1_{eg}$ | | | | | $1_{eg}$ | $1_{eg}$ | | | | | | | | 1 | 1 | |
| | GGT->TCC | 257 Gly->Ser | 56 | | | | | | | | | | | | | | | | | | | | | | | | | | | | | | | 1 | | | | |
| | GGT->GCC | 257 Gly->Ala | 60 | | | | | | | | | | | | | | | | | | | | $1_g$ | | | | | | | | | | | | | | | |
| | GGT->GCT | 257 Gly->Ala | 60 | | | | | | | | | | | | | | | | | | | | | 1 | | | | | | | | | | | | | | |
| | GGT->CAT | 257 Gly->His | 98 | | | | | | | | | | | | | | | | | | 1 | | | | | | | | | | | | | | | | | |
| | GGT->CGC | 257 Gly->Arg | 125 | | | | | | | | | | | | | | | | | | | | | | $1_g$ | | | | | | | | | | | | | 1 |
| | GGT->AGA | 257 Gly->Arg | 125 | | | | | | | | | | | | | | $1_g$ | $1_g$ | $1_{eg}$ | | | | | | | | | | | $1_g$ | $1_g$ | | | | | | | |
| TM6 | GTC->ATA | 278 Val->Ile | 29 | 0 | 0 | 0 | 0 | 0 | 0 | 0 | | 0 | | | | | | | | | | 1 | 1 | 1 | | | | | | | | | | | | | | |
| | GTC->ATT | 278 Val->Ile | 29 | | | | | | | | | | | 1 | 1 | 1 | 1 | | | 1 | 1 | | | | | 1 | | | 1 | 1 | 1 | | 1 | | 1 | | | 1 |
| | GTC->ATC | 278 Val->Ile | 29 | | | | | | | | | | 1 | | | | | 1 | 1 | | | | | | | | 1 | 1 | | | | 1 | | 1 | | 1 | | |
| | GTC->GTA | 278 Val->Ile | 29 | | | | | | | | 1 | | | | | | | | | | | | | | | | | | | | | | | | | | | |
| | GTC->GCT | 278 Val->Ala | 64 | | | | | | | | | | | | | | | | | | | | | | 1 | | | | | | | | | | | | | |
| C-terminal | GGA->AGT | 345 Gly->Ser | 56 | $0_j$ | $0_j$ | 0 | 0 | 0 | 0 | 0 | | | | | | | | | | | | | | | | 0 | 1 | 1 | | | | | | | | | | |
| | GGA->AGC | 345 Gly->Ser | 56 | | | | | | | | | | | | | | $1_j$ | | | | | | | | | | | | | | | | | | | | | |
| | GGA->GGC | 345 Gly->Ser | 56 | | | | | | | | | | | | | | | $1_j$ | | | | | | | | | | | | | | | | | | | | |
| | GGA->ACG | 345 Gly->Thr | 59 | | | | | | | | | | | | | | | | | | | $1_n$ | | | | | | | | | | | | | | | | |
| | GGA->GCA | 345 Gly->Ala | 60 | | | | | | | | | | | | | | | | | | | | | | | | | | | | | | | | | | $1_j$ | |
| | GGA->GCC | 345 Gly->Ala | 60 | | | | | | | | | | | | | | | | | | | | | | | | | | | | | | | | | | | 1 |
| | GGA->GTA | 345 Gly->Val | 109 | | | | | | | | | | | | | | | | 1 | | | | | | | | | | | | | | | | | | | |
| | GGA->AGG | 345 Gly->Arg | 125 | | | | | | | | $1_a$ | $1_{aj}$ | $1_{aj}$ | 1 | 1 | $1_{ai}$ | | | | 1 | $1_a$ | | $1_a$ | $1_a$ | $1_{aj}$ | | | | | $1_a$ | $1_a$ | $1_a$ | $1_j$ | $1_a$ | $1_a$ | $1_a$ | | |
| | GGA->AAA | 345 Gly->Lys | 127 | | | | | | | | | | | | | | | | | | | | | | | | | | $1_{ah}$ | | | | | | | | | |

Sites identified in the Bayes Empirical Bayes (BEB) analysis with a probability higher than 65% of being under ω > 1, which indicates positive selection. + Positively selected sites (*p* >95%); bold text indicates positively selected sites (*p* >99%). AVPR2 regions: ICL 3 = Intracellular 3; TM 6 = Transmembrane 6; and C-terminal = Carboxy-terminus. Zero (0) indicates the amino acid present in the *Homo sapiens* reference sequence, whereas 1 indicates a variant amino acid.

The nature of the modification does not represent an ancestry and descendant relationship. Grantham scores are predicted as conservative (0–50), moderately conservative (51–100), moderately radical (101–150), or radical (>151). Subscripted letters indicate the predicted presence of specific Eukaryotic Linear Motifs (as depicted using the code shown in Table S11). Scientific names of the species can be found in Table S1.
