# Supplementary material for: Oxytocin and arginine vasopressin receptor evolution: implications for adaptive novelties in placental mammals
Source: Genet Mol Biol. 2016 Aug 8;39(4):646–57. doi: 10.1590/1678-4685-GMB-2015-0323 (PMC5127151; doi:10.1590/1678-4685-GMB-2015-0323)
Supplement: Supplementary file 10 [file 1415-4757-gmb-1678-4685-GMB-2015-0323-Suppl11.pdf]

Table S11

Short linear interaction motifs (SLiMs) predicted for AVPR1a, AVPR1b, and AVPR2.

Functional motif class	Functional motif description	Code	Literature descriptions *
CLV_NRD_NRD_1	(NRD cleavage site): N-arginine dibasic convertase (NRD convertase) is an endopeptidase in dibasic site-processing secreted proteins.	a	N-Arg dibasic convertase is a metaloendopeptidase from the rat brain cortex and testis that cleaves peptide substrates on the N terminus of Arg residues in dibasic stretches (Pierotti et al. 1994).
CLV_PCSK_FUR_1	(PCSK cleavage site): The subtilisin-like proprotein convertases are expressed extensively in mammalian neural and endocrine cells and play a major role in the proteolytic processing of both neuropeptide and peptide hormone precursors.	b	This family appears to play an important role in neuroendocrine precursor processing (Rouillé et al. 1995).
MOD_LATS_1	(LATS kinase phosphorylation motif): The LATS/NDR kinases are an evolutionarily conserved subfamily of the basophilic AGC kinases. The two mammalian large tumor suppressors, LATS1/2, are collectively referred to as LATS and are highly conserved within their orthologous sets. LATS kinases were initially identified in Drosophila and named WARTS. The LATS act as key components of the Hippo-LATS pathway. At the core of this pathway in flies is the Ste-20-like kinase, Hpo, which phosphorylates and activates the NDR family kinase Lats/Warts in complex with Mats/dMOB1. The actived Mats/Lats complex then in turn phosphorylates its downstream effectors and inhibits their activity as transcriptional co-activators in *Drosophila*. Studies have shown that this pathway is highly conserved in mammals. The LATS phosphorylation site comprises a serine/threonine residue with a histidine at the −5 position.	c	LATS (large tumor suppressor) or warts is a Ser/Thr kinase that belongs to the Ndr/LATS subfamily of AGC (protein kinase A/PKG/PKC) kinases. It is a tumor suppressor gene originally isolated from Drosophila and recently isolated from mice and humans (Hao et al. 2008).
TRG_ER_diArg_1	(di Arginine retention/retrieving signal): The di-Arg retrieval and retention motif is present on membrane proteins where it serves for ER localization. A variety of membrane proteins (some multimeric) possess this di-Arg motif. The motif functions as a quality control mechanism for correct folding and protein complex assembly governing the ER exit. The functional motif needs to be exposed within a cytosolic region of the membrane protein and requires a distinct proximity to the transmembrane region. Heteromerization, as well as the interaction with 14-3-3 proteins or PDZ domain containing proteins can render some di-Arg retention signals inactive, whereas the interaction with Coat protein complex I (COPI) supports ER retrieval. Finally, some di-Arg based ER-retention signals may be negatively regulated by the phosphorylation of nearby residues.	d	The di-Arg retrieval and retention motif is involved in intracellular trafficking through the ER (Zerangue et al. 1999).
LIG_14-3-3_3	Consensus derived from reported natural interactors which do not match the Mode 1 and Mode 2 ligands. Key conserved residues are missing. While the sequence range of 14-3-3 binders is certainly not fully defined, a pattern derived from outliers as described here may be poorly predictive and matches should be treated with CAUTION. Validation is paramount.	e	The 14-3-3 motif family was suggested to participate in the complex signal for heterodimerization of GABA (B) R1 and GABA (B) R2 (Couve et al. 2001).
LIG_TRAF6	TRAF6 binding site. Members of the tumor necrosis factor receptor (TNFR) superfamily initiate intracellular signaling by recruiting the C-domain of TNFR-associated factors (TRAFs) through their cytoplasmic tails.	f	Cytosolic proteins that are recruited to membrane-associated receptors. ELMS LIG_TRAF2_1 were specifically found in GPCRs (Tovo-Rodrigues et al. 2014).
MOD_CK2_1	The main determinant of CK2 phosphorylation specificity is a negative charge 3 positions after the modification residue.	g	Protein kinase CK2 (also termed casein kinase-2 or -II) is a ubiquitous Ser/Thr-specific protein kinase required for viability and for cell cycle progression (Pinna and Meggio 1997).<br>In GPCR, this motif contributes to β-arrestin2 binding and trafficking along the recycling endosomal pathway (Lukashova et al. 2011).
CLV_PCSK_PC1ET2_1	The subtilisin-like proprotein convertases are expressed extensively in mammalian neural and endocrine cells and play a major role in the proteolytic processing of both neuropeptide and peptide hormone precursors.	h	This family appears to play an important role in neuroendocrine precursor processing (Rouillé et al. 1995).
DOC_MAPK_1	The docking interaction in the MAP kinase cascade is achieved through specific conserved regions on MAPKs (docking groove) and MAPK-interacting molecules (MAPK docking motif). The docking motif is usually - but not always - the sequence of a substrate protein.	i	Regulates processes such as cell proliferation, cell differentiation, and cell death in eukaryotes from yeast to humans.<br>GPCRs constitute important regulators of this family (Morrison 2012).
DOC_WW_Pin1_4	WW domains are small but abundant domains found in diverse regulatory situations. The binding peptide motifs appear always to involve proline residues. Specific motifs vary for different WW domains and in some cases must be phosphorylated on a serine or threonine.	j	A highly conserved domain of modular intracellular proteins, signal transduction proteins, and gene products interacting with the transcription machinery (Hofmann and Bucher 1995).
MOD_CK1_1	Motif recognized by CK1 for Ser/Thr phosphorylation.	k	The Casein kinase 1 family of protein kinases are serine/threonine-selective enzymes that function as regulators of signal transduction pathways in most eukaryotic cell types. CK1 isoforms are involved in Wnt signaling, circadian rhythms, nucleo-cytoplasmic shuttling of transcription factors, DNA repair, and DNA transcription (Eide and Virshup 2001).
MOD_GSK3_1	Site recognized by GSK3 for Ser/Thr phosphorylation.	l	GSK3 was identified as a protein kinase that is able to selectively regulate GPCRs (Chen et al. 2009).

MOD_ProDKin_1	Site at which MAP kinase phosphorylates substrates.	m	Mitogen-activated protein (MAP) kinases comprise a family of ubiquitous proline-directed, protein-serine/threonine kinases, which participate in signal transduction pathways that control intracellular events including acute responses to hormones and major developmental changes in organisms (Pearson et al. 2001).<br><br>GPCR kinases (GRKs) are best known for their role in homologous desensitization of GPCRs. GRKs phosphorylate activated receptors and promote high affinity binding of arrestins, which precludes G protein coupling (Gurevich 2012).
MOD_GlcNHglycan	Proteoglycans are extracellular proteins with glycosaminoglycan chains attached at a serine residue.	n	Proteoglycans and their glycosaminoglycan chains are important complex carbohydrates which regulates proteins in various partners. The glycosaminoglycans can assist GPCR binding to a protein (Lau et al. 2004).
MOD_PLK	Site recognized and phosphorylated by the Polo-like kinase.	o	There are indirect interaction description between Polo-like kinase and GPCRs, as it is reported to interact with GPCR Kinases (GRKs; (So et al. 2012).

All the above-described motifs have been confirmed by experimental data (http://elm.eu.org/).
* Description of protein involvement with G-protein coupled receptor (GPCR) signaling and/or the neuroendocrine system.

References

Chen L, Salinas GD, Li X (2009) Regulation of serotonin 1B receptor by glycogen synthase kinase-3. Mol Pharmacol 76:1150
Couve A, Kittler JT, Uren JM, Calver AR, Pangalos MN, Walsh FS, Moss SJ (2001) Association of GABA(B) receptors and members of the 14-3-3 family of signaling proteins. Mol Cell Neurosci 17:317
Eide EJ, Virshup DM (2001) Casein kinase I: another cog in the circadian clockworks. Chronobiol Int 18:389
Gurevich EV (2012) G protein-coupled receptor kinases: More than just kinases and not only for GPCRs. In: Tesmer JJG (ed)
Hao Y, Chun A, Cheung K, Rashidi B, Yang X (2008) Tumor suppressor LATS1 is a negative regulator of oncogene YAP. J Biol Chem 283:5496
Hofmann K, Bucher P (1995) The rsp5-domain is shared by proteins of diverse functions. FEBS Lett 358:153
Lau EK, Paavola CD, Johnson Z, Gaudry JP, Geretti E, Borlat F, Kungl AJ, Proudfoot AE, Handel TM (2004) Identification of the glycosaminoglycan binding site of the CC chemokine, MCP-1: implications for structure and function in vivo. J Biol Chem 279:22294
Lukashova V, Szabó EZ, Jinadasa T, Mokhov A, Litchfield DW, Orlowski J (2011) CK2 phosphorylation of an acidic Ser/Thr di-isoleucine motif in the Na+/H+ exchanger NHE5 isoform promotes association with beta-arrestin2 and endocytosis. J Biol Chem 286:11456
Morrison DK (2012) MAP kinase pathways. Cold Spring Harb Perspect Biol 4
Pearson G, Robinson F, Beers Gibson T, Xu BE, Karandikar M, Berman K, Cobb MH (2001) Mitogen-activated protein (MAP) kinase pathways: regulation and physiological functions. Endocr Rev 22:153
Pierotti AR, Prat A, Chesneau V, Gaudoux F, Leseney AM, Foulon T, Cohen P (1994) N-arginine dibasic convertase, a metalloendopeptidase as a prototype of a class of processing enzymes. Proc Natl Acad Sci U S A 91:6078
Pinna LA, Meggio F (1997) Protein kinase CK2 ("casein kinase-2") and its implication in cell division and proliferation. Prog Cell Cycle Res 3:77
Rouillé Y, Duguay SJ, Lund K, Furuta M, Gong Q, Lipkind G, Oliva AA, Chan SJ, Steiner DF (1995) Proteolytic processing mechanisms in the biosynthesis of neuroendocrine peptides: the subtilisin-like proprotein convertases. Front Neuroendocrinol 16:322
So CH, Michal AM, Mashayekhi R, Benovic JL (2012) G protein-coupled receptor kinase 5 phosphorylates nucleophosmin and regulates cell sensitivity to polo-like kinase 1 inhibition. J Biol Chem 287:17088
Tovo-Rodrigues L, Roux A, Hutz MH, Rohde LA, Woods AS (2014) Functional characterization of G-protein-coupled receptors: A bioinformatics approach. Neuroscience 277:764
Zerangue N, Schwappach B, Jan YN, Jan LY (1999) A new ER trafficking signal regulates the subunit stoichiometry of plasma membrane K(ATP) channels. Neuron 22:537
